# The decline in postural balance has a negative impact on the performance of functional tasks in individuals with Parkinson's Disease

**DOI:** 10.1016/j.clinsp.2024.100382

**Published:** 2024-05-16

**Authors:** Natália Mariana Silva Luna, Tatiana Godoy Bobbio, Myriam de Graaf, Júlia Maria D'Andrea Greve, Rita de Cássia Ernandes, Aluane Silva Dias, Matheus Henrique dos Santos Lino, Jose Maria Soares-Junior, Edmund Chada Baracat, Luis Mochizuki, Guilherme Carlos Brech, Angelica Castilho Alonso

**Affiliations:** a Program in Aging Sciences from the Universidade São Judas Tadeu (USJT), São Paulo, SP, Brazil; b Laboratory for the Study of Movement, Institute of Orthopedics and Traumatology, Faculdade de Medicina da Universidade de São Paulo (FMUSP), São Paulo, SP, Brazil; c University of St. Augustine for Health Sciences, Miami Campus, Miami, United States of America; d Movement Science, University of Münster, Münster, Germany; e Otto Creutzfeldt Center for Cognitive and Behavioral Neuroscience, University of Münster, Münster, Germany; f Discipline of Gynecology, Department of Obstetrics and Gynecology, Hospital das Clínicas, Faculdade de Medicina da Universidade de São Paulo (FMUSP), São Paulo, SP, Brazil; g School of Arts, Sciences and Humanities, Universidade de São Paulo, São Paulo, SP, Brazil

**Keywords:** Aging, Parkinson's disease, Postural balance, Accidental falls, Functional Mobility

## Abstract

**Introduction:**

An accurate assessment of balance problems is critical for decreasing the risk of falling in patients with Parkinson's Disease (PD). Reliable diagnostic tools such as Computerized Dynamic Posturography (CDP) are not feasible for the clinical setting. Therefore, the present study's aim was to assess the correlation between the clinical Balance Evaluation Systems Test (BESTest) and CDP.

**Methods:**

20 male older adults with Parkinson's Disease (PD) were included in this study. Participants first executed the Sit-To-Stand (STS), Step/Quick turn (SQT), and Step Up and Over (SUO) tests on a Balance Master® force platform, followed by a clinical balance evaluation using the BESTest.

**Results:**

Four outcomes of the CDP were negatively correlated with one or more BESTest domains or total BESTest score: STS sway velocity was negatively correlated with the anticipatory postural adjustment (*p* = 0.02) and sensory orientation (*p* = 0.01) domains. SQT turn time was negatively correlated with biomechanical restriction (for turns to the left, *p* = 0.01, and right, *p* = 0.03, respectively), postural response (*p* = 0.01, *p* = 0.01), dynamic balance during gait (*p* = 0.007, *p* = 0.001), and total score (*p* = 0.02, *p* = 0.01). Step over time to the right in SUP was negatively correlated with the limits of the stability domain (*p* = 0.002) and total BESTest score (*p* = 0.020). SUO impact index was negatively correlated with the anticipatory postural adjustment domain (*p* = 0.01).

**Conclusion:**

This study shows that several BESTest domains are significantly correlated with CDP outcomes, demonstrating that the BESTest can be used as a more clinically feasible alternative for computerized posturography, without loss of information.

## Highlights

• Balance impacts functional tasks in Parkinson's disease patients.
• Specific treatment for balance deficits in Parkinson's disease patients.
• BESTest × force platforms.

## Introduction

Parkinson's Disease (PD) is one of the most important chronic diseases that arise during the aging process, due to the gravity of its symptoms and its high prevalence in elders.^1^ PD is characterized by a progressive degeneration of dopaminergic neurons of the *substantia nigra*, located in the compact zone of the mesencephalon.^2^ Motor signs in PD include resting tremor, muscular rigidity, freezing of gait, bradykinesia, a shuffling gait, and postural instability.^3^

As PD develops, there is a progressive worsening of postural balance, which is associated with falls, limited mobility, and loss of independence.^4^ These symptoms make balance an important issue to target in rehabilitation. To effectively improve balance – or to prevent a regression in balance – it is important to tailor the rehabilitation to the needs of the individual. For this, an accurate assessment of balance is needed.^5^

Computerized Dynamic Posturography (CDP) is a reliable method to assess balance control and to evaluate the integration of the visual, somatosensory, and vestibular systems in maintaining balance. In CDP, balance test protocols, such as sensory interaction on balance, perturbed balance control, and limits of stability, are performed on a force platform, giving feedback on postural instability and which sensory dysfunctions could be associated with any discovered imbalance. Although CDP is a reliable method for cataloging postural instability, for health care providers, for example, its use is limited due to high costs and limited portability.^6^^,^^7^

Functional balance tests, like the BESTest, are a feasible alternative to evaluate balance for health care providers. The BESTest shower specificity 0.92, the results corroborate the diagnostic and screening functions of the BESTest and indicate that the Brazilian versions exhibit adequate reliability, construct validity, response stability, and capacity to distinguish among various balance ability levels in older adults and individuals with PD.^8^ The BESTest items are clustered in sections corresponding to the six underlying systems that may constrain balance: biomechanical, stability limits, postural responses, anticipatory postural adjustments, sensory orientation, and dynamic balance during gait.^8^^,^^9^

For persons with PD, it is not clear how functional tests, like the BESTest, and biomechanical tests can be combined to understand the PD's postural issues. In fact, to properly tailor the balance interventions during rehabilitation, therapists need to be able to detect the postural instabilities in PD to decrease the risk of falling. Therefore, the aim of this study was to investigate the correlation between Computerized Posturography and BESTest evaluation in persons with Parkinson's disease. Correlations between the two test outcomes can help clinicians.

Therefore, the aim of this study was to investigate the correlation between systems that involve postural balance, as tested with CDP, and functional tasks, as documented by the BESTest. Correlations between the two test outcomes can help clinicians gain more information from the BESTest, without needing expensive force platforms.

## Methods

This cross-sectional study was approved by the Research Ethics Committee of the University of São Judas Tadeu (CAAE 53,406,116.2.0000.0089). It was developed at the Laboratory for the Study of Movement, Institute of Orthopedics and Traumatology, School of Medicine at the Universidade de São Paulo (FMUSP) and the Universidade São Judas Tadeu (USJT), São Paulo, SP, Brazil.

### Participants

It was a convenience sample, with twenty (*n* = 20) persons with PD with a media age of (67.1 ± 5.8 years-old) were included in this study. The inclusion criteria were: a Mini-Mental State Examination (MMSE)^10^ score ≥ 24; no other diseases, injuries or surgeries to the musculoskeletal system that could influence mobility; and no concomitant treatment with experimental drugs. The participants had to show a PD diagnosis with stages 1 to 3 on the Hoehn and Yahr modified Scale (H & Y Scale).^11^ Candidates were selected to participate in this study from the Neurology Service at the Hospital das Clinicas (HC) ‒ FMUSP. The participant's demographic characteristics are described in Table 1.

**Table 1:** Demographic Characteristics of subjects with PD.

	M (SD)
**Age (years)**	67.1 (5.8)
**Weight (kg)**	63.0 (8.8)
**Height (cm)**	160 (5.4)
**Years of diagnostic (years)**	11.5 (5.7)
**UPDRS – motor score**	9.6 (2.5)
**Hoehn & Yahr**	2.5 (4.2)

UPDRS, Unified Parkinson's Disease Rating Scale.

### Measures

For all participants, balance control was tested in two ways: first via Computerized Dynamic Posturography (CDP), and secondly with the clinical BESTest. Additionally, disease severity was assessed using the motor section III for the Unified Parkinson's Disease Rating Scale (UPDRS) during the “on” period of the disease.

#### Computerized dynamic posturography

The balance control evaluation was performed on the NeuroCom Balance Master® force platform system (NeuroCom International, Inc., Clackamas, OR, USA). This is a dual^−^force plate system that can be used to measure the vertical forces exerted by the patient's feet.^10^ Three test protocols were selected for this study, namely: sit to stand; step/quick turn; and step up and over. For all three protocols, three trials were conducted.^11^

1) Sit-to-Stand Test (STS): The patient started from a seated position on a bench without a backrest or arm support, with knees flexed 90° and heels 10 cm apart. Upon visual and auditory instruction (a green signal at the top of the screen plus a verbal command), the participant had to stand up from the bench and hold steady, looking at a fixed point at eye level, with the arms relaxed beside the body. During the test, the weight transfer time (time required to transfer from the buttock to the feet), rising index (vertical force required to rise), weight symmetry (the symmetry between the left and right legs during rising), and COG sway velocity were recorded.^7^^,^^11^
2) Step/Quick Turn (SQT): In this test, the patient started in a standing position and was instructed to take two steps forward, turn 180°, and return to the start location. This task was repeated six times (3 to the left and 3 to the right). The turn-time and turn-sway velocity were recorded.^7^^,^^11^
3) Step Up and Over (SUO): The patient started in a standing position. Upon a visual signal (green light) and a verbal command (“climb up”), the participant stepped forward onto a curb with the left foot, lifting the body up and over the curb, to step down with the right foot on the other side of the curb. The movement was finished by placing the left foot parallel to the right food on the other side of the curb. The participant was not allowed to touch the curb with the swing leg. After three trials with one foot, the order was reversed. The measured parameters were the lift-up index (maximum vertical force exerted by the step-up leg), movement time (average time needed to complete the step-over), and the impact index (average maximum vertical force during landing of the swing leg).^7^^,^^11^

#### Balance evaluation systems test (BESTest)

Static and dynamic clinical balance assessments were performed using the BESTest. The BESTest is a 36-item clinical assessment tool, developed to assess balance across six domains of postural control: biomechanical constraints; limits of stability; postural response to induced loss of balance; anticipatory postural adjustments; sensory orientation; and dynamic balance during gait. Scoring of each item is done on a range of 0 (severe impairment) to 3 (no impairment).^6^^,^^12^

#### Assessment of motor function: unified parkinson's disease rating scale (UPDRS)

Motor function was assessed using the Unified Parkinson's Disease Rating Scale (UPDRS), which is an instrument/scale with excellent reliability (*r* = 0.96) and validity (convergent validity and criteria-related), qualifying it as a good method for evaluating PD (section III).^13^

### Statistical analysis

All statistical analyses were conducted using SPSS for Windows 20.0 and statistical significance was set at *p* < 0.05. For all variables, normality was checked using the Kolmogorov-Smirnov Test and descriptive statistics (mean, standard deviation and frequencies) were calculated. The Pearson correlation test was applied to measure the correlation between the outcome variables of all three CDP tests and the BESTest domains.

## Results

Table 2 shows all results for the correlation analyses between CDP outcome variables and the BESTest scores.

**Table 2:** Correlation between BESTest and functional tasks in elderly with DP.

	Biomechanical restriction r(p)	Limits of Stability r(p)	Transitions/ Anticipatory r(p)	Reactive postural r(p)	Sensory Orientation r(p)	Gait Stability r(p)	Total Score r(p)
**STEP/QUICK TURN**							
**Turn Time L (s)**	−0.53 (0.01)*	−0.24 (0.32)	−0.53 (0.01)*	−0.22 (0.353)	−0.41 (0.08)	−0.60 (0.007)^⁎⁎^	−0.52 (0.022)*
**Turn Time R(s)**	−0.48 (0.03)*	−0.04 (0.84)	−0.56 (0.01)*	−0.41 (0.07)	−0.40 (0.08)	−0.70 (0.001)^⁎⁎^	−0.54 (0.01)*
**Turn Sway L (°/s)**	−0.18 (0.44)	−0.08 (0.73)	0.05 (0.82)	0.01 (0.95)	0.10 (0.68)	−0.40 (0.07)	−0.07 (0.75)
**Turn Sway R (°/s)**	−0.14 (0.56)	0.09 (0.69)	−0.20 (0.41)	−0.24 (0.31)	0.09 (0.69)	−0.57 (0.01)*	−0.17 (0.48)
**SIT TO STAND**							
**WT Transfer (s)**	−0.32 (0.18)	−0.19 (0.42)	−0.20 (0.40)	0.22 (0.35)	−0.032 (0.89)	−0.02 (0.92)	−0.10 (0.67)
**Rising index (%)**	−0.18 (0.44)	−0.24 (0.30)	−0.25 (0.30)	−0.51 (0.02)*	−0.55 (0.01)*	−0.20 (0.39)	−0.44 (0.06)
**COG Sway (*°*/s)**	−0.08 (0.72)	0.04 (0.86)	0.02 (0.91)	−0.01 (0.97)	−0.11 (0.62)	0.06 (0.80)	−0.03 (0.90)
**STEP UP AND OVER**							
**Lift Up Index L** (%)	0.10 (0.69)	0.17 (0.51)	0.07 (0.77)	−0.11 (0.68)	−0.10 (0.70)	0.13 (0.60)	0.04 (0.86)
**Lift Up Index R** (%)	−0.11 (0.66)	−0.05 (0.84)	0.02 (0.93)	−0.24 (0.36)	−0.30 (0.24)	0.01 (0.95)	−0.18 (0.50)
**Movement Time L**(s)	−0.242 (0.36)	−0.44 (0.08)	−0.230 (0.39)	−0.143 (0.59)	−0.023 (0.93)	−0.275 (0.30)	−0.286 (0.28)
**Movement Time R** (s)	−0.41 (0.11)	−0.72 (0.002)*	−0.49 (0.05)	−0.27 (0.30)	−0.30 (0.25)	−0.34 (0.19)	−0.57 (0.02)*
**Impact Index L** (%)	−0.02 (0.92)	−0.07 (0.78)	−0.17 (0.51)	−0.58 (0.01)*	−0.29 (0.27)	0.21 (0.43)	−0.25 (0.35)
**Impact Index R** (%)	0.06 (0.82)	0.04 (0.98)	−0.06 (0.82)	−0.25 (0.25)	−0.26 (0.31)	0.09 (0.73)	−0.12 (0.64)

L, Left; R, Right; WT, Weight Transfer; COG, Center of Gravity.

For the STS test, a negative correlation was found between sway velocity in standing position and two BESTest domains: anticipatory postural adjustment *p* = 0.02 and sensory orientation *p* = 0.01).

For the SQT test, a negative correlation was found between turn time to both sides (Left, L, and Right, R) and several BESTest domains: biomechanical restriction (L: *p* = 0.01, and R: *p* = 0.03), postural response (L: *p* = 0.01, and R: *p* = 0.01), dynamic balance during gait (L: *p* = 0.007, and R: *p* = 0.001), and total score (L: *p* = 0.02, and R: *p* = 0.01).

For the SUO test, a negative correlation was found between movement time to complete the step over with the right side first and BESTest limits of the stability domain (*p* = 0.002) and the total score (*p* = 0.02), as well as between impact index and the anticipatory postural adjustment domain (*p* = 0.01).

## Discussion

The main finding of the study is that functional tasks such as walking and turning, climbing and descending stairs, and sitting and standing are correlated with different balance control domains, defined by the BESTest. These findings can be helpful in tailoring rehabilitation programs for people with PD.

Biomechanical restrictions can impair a walking and turning task. The score on the biomechanical restrictions BESTest domain was negatively correlated with the time to perform the walking and turning (SQT) task. This relationship is linked to the quality of the foot support base, hypomobility (due to PD) leading to postural misalignment, due to the hypertonic activation of the muscles of the feet. lower limbs that decrease the ability to generate strength in the functional movement of the ankle and hip used to stand.^14^

The delay in anticipatory adjustments also increased the time to complete the task, corroborating other studies^5^^,^^15^^,^^16^ that assessed postural balance and showed that elderly people with PD are able to maintain a static erect posture with less oscillation of the center of mass of the body in face of a postural transition that involves repetitive weight change in the lower limbs, which characterizes lower chances of performing anticipatory postural strategies and, therefore, a greater risk of falls.

Decreased dynamic balance during gait and the total test score (BESTest) increased the time to perform the task, this was due to the difficulty of turning over for the elderly with PD, which is slower and reveals a narrower support base, consequently a greater number of steps (small distance between the feet) is needed compared to healthy elderly people. This increased task time and balance at the end of the test. Other authors^17^^,^^18^ state that the elderly with PD are slower to turn to compensate for postural instability. The disease compromises the ability to initiate and organize motor commands, which directly impacts the ability to turn at high speed or even perform some tasks.^18^

In the sitting and standing tasks, the decrease in reactive postural response (balance strategies) and sensory orientation increased the rising index (force exerted to rise). During this task in normal individuals, the center of gravity moves anteriorly and needs to be controlled through concentric and eccentric contraction of the lower limb muscles, in order to promote postural adjustment and lead to a stable position on a bipedal basis,^19^^,^^20^ different from it occurs in elderly people with PD, where there is a delay in adjustment, due to a failure in automatic motor control that requires rapid muscle response.^15^

In patients with PD, this control is hampered by deficits in muscle strength, deficits triggered not only by sarcomere losses, as in biological aging, but also by cytoplasmic inclusions, such as Lewy bodies in muscle cells; and membrane disruption, mitochondrial depolarization, cytoskeletal changes, and oxidative stress.^21^

The muscular impairment of PD significantly affects the ability to recruit motor units, and therefore the control needed for agonist-antagonist synergy and as a compensation strategy increases the strength of the lower limbs to push the platform. Further, individuals with PD have difficulty maintaining steady upright posture and responding to external and internal perturbations.^12^^,^^14^

Decreasing stability limits and total score increases the time to perform the task of going up and down stairs. PD patients have lower COP oscillation^18^^,^^22^ and lower stability limits (maximum swing of the center of gravity) that are related to postural inflexibility.^14^

A lower stability limit promotes impairments in postural corrections during the task of going up and down stairs and thus increases the task execution time.^23^ Furthermore, correlations are found between the step-over-obstacle task and executive function. This task requires executive function (problem-solving, sequencing, shifting attention), which is affected by PD.^24^

In this same task, elderly people with PD unloaded more body weight on the left side (Impact Index L) when they presented worse postural reactive. It is suggested that PD postural instability triggers poor anticipatory control.^25^ That alters weight bearing during dynamic tasks. Other examinations have revealed individuals with PD have difficulty sequencing motor programs for postural correction during perturbations.^12^ Similarly, the underlying problem of akinesia is an inability to drive the necessary motor output to a complete complex task.^26^

The disease causes damage to motor sequence components (e.g., weight transfer difficulty) and risk of falling^27^ and the greatest impact force on one side of the limbs during the task would have been indicative of a compensatory strategy to decrease *tal risco* ‒ the risk of falling.^27^ The ability to negotiate obstacles in the environment is required for independent living. This task may become particularly difficult as strength and postural stability are compromised during the aging process and/or in a progressive disease state such as in Parkinson's Disease (PD). Because a trip is one of the most common contributors to a fall,^28^ understanding the movement characteristics of stepping up and over an obstacle is critical for fall prevention and interventions in individuals with Parkinson's disease.

The clinical implications of the study are related to the contribution to a better knowledge of the disease in relation to functionality, pointing to possible guidelines on how to conduct an exercise program that can improve the functional activities of elderly people with PD. Including an exercise routine that improves biomechanical restrictions, especially those of ankle, knee and hip, stability limits, postural responses, anticipatory postural adjustments, sensory orientation, and dynamic balance during gait will make elderly people with PD more confident to perform functional tasks. Leaving them more confident and prepared to act more assertively.

In this study, sample size calculation was not performed, which may be a limitation of the study, the sample size. In addition, evaluating each postural balance system accurately and reliably in a computerized and segmented way still makes this a limitation of biomechanical studies, it is not possible to differentiate the systems within the same evaluation.

## Conclusion

The worst performance in systems involving postural balance negatively impacts the functional tasks of daily life in individuals with PD. The study demonstrates the need to assess systems as a way of contributing to the specific treatment of balance deficits in elderly people with PD.

## Conflicts of interest

The authors declare no conflicts of interest.

## References

[1] Rajput A.H., Rajput E.F. (2017). Octogenarian parkinsonism – Clinicopathological observations. Parkinsonism Relat Disord.

[2] Frazzitta G., Maestri R., Uccellini D., Bertotti G., Abelli P. (2009). Rehabilitation treatment of gait in patients with Parkinson's disease with freezing: a comparison between two physical therapy protocols using visual and auditory cues with or without treadmill training. Mov Disord.

[3] Mehdizadeh M., Martinez-Martin P., Amirhasan Habibi S., Nikbakht N., Alvandi F., Bazipoor P. (2019). The association of balance, fear of falling, and daily activities with drug phases and severity of disease in patients with Parkinson. Basic Clin Neurosci.

[4] Pasluosta C., Hannink J., Gaßner H., Von Tscharner V., Winkler J., Klucken J. (2018). Motor output complexity in Parkinson's disease during quiet standing and walking: analysis of short-term correlations using the entropic half-life. Hum Mov Sci.

[5] Luna N.M.S., Brech G.C., Canonica A., Ernandes R de C., Bocalini D.S., Greve J.M.D.A. (2020). Effects of treadmill training on gait of elders with Parkinson's disease: a literature review. Einstein (Sao Paulo).

[6] Maia A.C., Rodrigues-de-Paula F., Magalhães L.C., Teixeira R.L.L. (2018). Cross-cultural adaptation and analysis of the psychometric properties of the Balance Evaluation Systems Test and MiniBESTest in the elderly and individuals with Parkinson's disease: application of the Rasch model. Braz J Phys Ther.

[7] Ernandes R de C., Brech G.C., Luna N.M.S., Nunes M.F., Greve J.M.D.A., Leme L.E.G. (2020). Relationship of force platform with the clinical balance evaluation systems test in older adults. Acta Ortop Bras.

[8] Folstein M.F., Folstein S.E., McHugh P.R. (1975). Mini-mental state”. A practical method for grading the cognitive state of patients for the clinician. J Psychiatr Res.

[9] Hoehn M.M., Yahr M.D. (1967). Parkinsonism: onset, progression, and mortality. Neurology.

[10] Larsen-Merrill J., Lazaro R (2008). Use of the neurocom balance master™ training protocols to improve functional performance in a person with multiple sclerosis. J student Phys therpy Res.

[11] Tomomitsu M.S .V, Alonso A.C., Morimoto E., Bobbio T.G., Greve J.M.D. (2013). Static and dynamic postural control in low-vision and normal-vision adults. Clinics (Sao Paulo).

[12] Horak F.B., Wrisley D.M., Frank J. (2009). The balance evaluation systems test (BESTest) to differentiate balance deficits. Phys Ther.

[13] Jenkins M.E., Johnson A.M., Holmes J.D., Stephenson F.F., Spaulding S.J. (2010). Predictive validity of the UPDRS postural stability score and the Functional Reach Test, when compared with ecologically valid reaching tasks. Park Relat Disord.

[14] Matsuda K., Suzuki Y., Yoshikawa N., Yamamoto T., Kiyono K., Tanahashi T. (2016). Postural flexibility during quiet standing in healthy elderly and patients with Parkinson's disease. Annu Int Conf IEEE Eng Med Biol Soc.

[15] Bronte-Stewart H.M., Minn A.Y., Rodrigues K., Buckley E.L., Nashner L.M. (2002). Postural instability in idiopathic Parkinson's disease: the role of medication and unilateral pallidotomy. Brain.

[16] D'Andréa Greve J.M., Luna N.M.S., De Siqueira J.P., Prota C., Alonso A.C. (2014). Assessment of postural balance among individuals with parkinson disease with and without effects from dopaminergic medications. Am J Phys Med Rehabil.

[17] Mellone S., Mancini M., King L.A., Horak F.B., Chiari L. (2016). The quality of turning in Parkinson's disease: a compensatory strategy to prevent postural instability?. J Neuroeng Rehabil.

[18] Rahal MA, Andrusaitis R, Rodrigues S, Alonso AC, Maria D’J, Greve A. et al. Gait, posture and transfer assessment among elderly practitioners and non-practitioners of Tai Chi Chuan. 2013;5(12A):117–21.

[19] Anto M., Ange I., Alonso C., Felix I.I., Andrusaitis R., Thuam I.I. (2015). Analysis of static and dynamic balance in healthy elderly practitioners of Tai Chi Chuan versus ball-room dancing. Clinics (Sao Paulo).

[20] Collier T.J., Kanaan N.M., Kordower J.H. (2017). Aging and Parkinson's disease: different sides of the same coin?. Mov Disord.

[21] Shen X., Wong-Yu I.S.K., Mak M.K.Y. (2016). Effects of exercise on falls, balance, and gait ability in Parkinson's disease: a meta-analysis. Neurorehabil Neural Repair.

[22] Barbosa A.F., De Souza C.O., Chen J., Francato D.V., Caromano F.A., Chien H.F. (2015). The competition with a concurrent cognitive task affects posturographic measures in patients with parkinson disease. Arq Neuropsiquiatr.

[23] Souza C de O., Voos M.C., Barbosa A.F., Chen J., Francato D.C.V., Milosevic M. (2019). Relationship between posturography, clinical balance and executive function in Parkinson´s disease. J Mot Behav.

[24] Adkin A.L., Frank J.S. (2003). Jog MS. Fear of falling and postural control in Parkinson's disease. Mov Disord.

[25] Marsden C.D., Obeso J.A. (1994). The functions of the basal ganglia and the paradox of stereotaxic surgery in Parkinson's disease. Brain.

[26] Nocera J.R., Price C., Fernandez H.H., Amano S., Vallabhajosula S., Okun M.S. (2010). Tests of dorsolateral frontal function correlate with objective tests of postural stability in early to moderate stage Parkinson's disease. Park Relat Disord.

[27] Berg W.P., Alessio H.M., Mills E.M., Tong C. (1997). Circumstances and consequences of falls in independent community-dwelling older adults. Age Ageing.

